# Altered Default Mode Network Dynamics in Civil Aviation Pilots

**DOI:** 10.3389/fnins.2019.01406

**Published:** 2020-01-14

**Authors:** Xi Chen, Kaijun Xu, Yong Yang, Quanchuan Wang, Hao Jiang, Xiangmei Guo, Xipeng Chen, Jiazhong Yang, Cheng Luo

**Affiliations:** ^1^Institute of Aviation Human Factors and Ergonomics, Department of Aviation Psychology, Institute of Flight Technology, Civil Aviation Flight University of China, Guanghan, China; ^2^Key Laboratory for NeuroInformation of Ministry of Education, School of Life Sciences and Technology, University of Electronic Science and Technology of China, Chengdu, China

**Keywords:** pilot, resting-state fMRI, functional connectivity, default mode network, flight hours

## Abstract

**Background:**

Airlines occupy an increasingly important place in the economy of many countries. Because air disasters may cause substantial losses, comprehensive surveys of the psychophysiological mechanism of flying are needed; however, relatively few studies have focused on pilots. The default mode network (DMN) is an important intrinsic connectivity network involved in a range of functions related to flying. This study aimed to examine functional properties of the DMN in pilots.

**Method:**

Resting-state functional magnetic resonance imaging data from 26 pilots and 24 controls were collected. Independent component analysis, a data-driven approach, was combined with functional connectivity analysis to investigate functional properties of the DMN in pilots.

**Results:**

The pilot group exhibited increased functional integration in the precuneus/posterior cingulate cortex (PCC) and left middle occipital gyrus. Subsequent functional connectivity analysis identified enhanced functional connection between the precuneus/PCC and medial superior frontal gyrus.

**Conclusion:**

The pilot group exhibited increased functional connections within the DMN. These findings highlight the importance of the DMN in the neurophysiological mechanism of flying.

## Introduction

Airlines continue to grow at a high speed and now occupy an increasingly important place in the economy of many countries. Due to the implementation of automated equipment, the pilot’s work is not to manually control flight, but to monitor the entire system in real time. Human factors in aviation accidents have become the main causes. It appears that the most effective strategies to promoting aviation safety is to select pilots with high safety capabilities. Objective indicators of safety capability are needed.

Pilot personality traits and social cognition variables have been frequently studied (Musson et al., 2004; Pauley et al., 2008; Poropat, 2009; You et al., 2013). However, the relationship between personality traits and safety capability was ambiguous. Some studies have focused on the neural mechanism of specific processes during flying such as decision making (Causse et al., 2013; Adamson et al., 2014) and mental strategy (Peres et al., 2000). Frontal regions were found to contribute to aeronautical decision making and aviation track-following task. FNIRS and EEG were also used to investigate the neural activity of working memory and the coordinated interaction of pilot under flight simulator and real flight conditions (Toppi et al., 2016; Gateau et al., 2018). Results showed pilots in flight condition had higher prefrontal cortex activation. In addition, during the higher level of cooperative flight phases, there was dense interbrain connectivity linking frontal and parietal brain areas. Glider flying requires pilots to control the aircraft at relatively high speeds in three dimensions, which are the same processes in airline flying (Callan et al., 2013; Durantin et al., 2017). The increased gray matter density of glider pilots in premotor cortex and anterior cingulate cortex was identified, which might be associated with cognitive and motor processes related to flying (Callan et al., 2013).

In general, the frontal cortex and motor related brain areas may involve in flying activity. However, the results are scattered and details regarding the flying-related mechanism remain unclear. More in-depth and comprehensive surveys of the psychophysiological mechanism(s) of flying are urgently needed.

Neuroimaging has been broadly used in the investigation of the human brain (Jiang et al., 2019a). In particular, resting-state functional magnetic resonance imaging (fMRI) is a very useful technique (Dong et al., 2016; Jiang et al., 2019b). The brain consumes 60–80% of the energy in the resting state; however, during task-related states, energy consumption rises only by approximately 0.5–1% (Raichle and Snyder, 2007). Unlike task-related fMRI which focus on the goal-directed brain activity, resting state represents the baseline state in the human brain. As such, intrinsic functioning during the rest state is important. In addition, the human brain is a highly interconnected complex patchwork, and network approaches may be useful for deeply understanding the specific brain organization pattern. The functional connectivity model during the resting state (the intrinsic connectivity network) reflects significant coupling of spontaneous fluctuations in ongoing activity and provide a neuroanatomical foundation for understanding human behavior and cognitive function. Thus, studying the intrinsic connectivity networks in pilots may reveal the essential features of flying and provide valuable insights to understanding the psychophysiological mechanism(s) of flying.

Among the many stable intrinsic connectivity networks identified in the human brain, the default mode network (DMN) has been assigned particular importance. The prefrontal cortex, precuneus/posterior cingulate cortex (PCC) and bilateral parietal cortex always exhibit high levels of activity and high degrees of functional connectivity during the resting state. These regions are considered the DMN (Uddin et al., 2009; Gong et al., 2019). The prefrontal cortex, which may involve in flying related activity, was one of the core areas of DMN. The DMN is related to different aspects of self-referential mental processes, such as value-based decision making (Rangel et al., 2008), episodic memory retrieval (Sestieri et al., 2011; Vannini et al., 2011) and visuospatial imagery (Cavanna and Trimble, 2006). It appears that functions, such as decision making (Causse et al., 2011, 2013) and visuo-spatial imagery (Piccardi et al., 2013), which are of vital importance in flying, are closely related to the DMN.

Given its potential roles, we focused on examining the functional properties of the DMN in pilots. In the current study, resting-state fMRI data from pilots and controls were collected. Independent component analysis (ICA) was used to decompose the data to reveal functional patterns of the DMN. ICA is a data-driven, multivariate method that can capture the entire DMN. We hypothesized that intrinsic DMN activity may exhibit different patterns in pilots and that these changes would be related to their total flight hours. These findings may contribute to mapping of the neural mechanism of flying to some degree.

## Materials and Methods

### Participants

In total, 26 pilots on active duty and 24 healthy controls participated in this study. Fourteen pilots were flight instructors from the Civil Aviation Flight University of China. Their aircraft types were C172R, PA44, SR-20, PA44, MA600, and CE525. Twelve pilots were first officers from different airlines. Their aircraft types were Boeing 737 or Airbus 320. The two groups were matched for sex, handedness, and education characteristics. Individuals with a history of neurological illness, traumatic brain injury, substance-related disorders, or standard contraindications to MRI were excluded from this study.

The experimental procedures (No. 2018-042002) were approved by the Ethics Committee of University of Electronic Science and Technology of China (Chengdu, China), and written informed consent was obtained from all participants.

### Data Acquisition

The MRI data were acquired using a 3-Tesla MRI scanner (DISCOVERY MR 750, GE Healthcare, Waukesha WI, United States) at the Center for Information in Medicine of University of Electronic Science and Technology of China. Subjects were scanned in a supine, head-first position. Cushions were placed on both sides of the head to decrease head motion.

High-spatial-resolution structural images were acquired using a T1-spoiled gradient recalled echo pulse sequence. The scan parameters were as follows: repetition time (TR), 5.976 ms; echo time (TE), 1.976 ms; flip angle, 9°; matrix, 256 × 256; slice thickness, 1 mm (no gap); field of view (FOV), 25.6 cm × 25.6 cm; and slice number, 154.

A gradient-echo echo-planar imaging sequence was used to collect resting-state fMRI data. The participants were instructed to lying quietly and close their eyes without falling asleep during scanning. A period of 510 s resting-state functional images were also collected for each subject. The scan parameters were as follows: TR, 2000 ms; TE, 30 ms; flip angle, 90°; matrix, 64 × 64; FOV, 24 cm × 24 cm, slice thickness, 4 mm (no gap); slice number, 35 (each volume). In total, 255 volumes were acquired.

### Data Preprocessing

Image pre-processing was based on the SPM12 (Statistical Parametric Mapping 12) toolbox^1^. For resting-state fMRI data, the first five scans of each subject were discarded for magnetization equilibrium. The remaining 250 images were slice-time and head-motion corrected. One pilot was excluded from the analyses due to excessive head motion (a maximum displacement exceeding 2 mm in any direction or a maximum spin exceeding 2°). Demographic characteristics of the two groups are summarized in Table 1. Head motion differences between the groups were compared. In addition, the head motion of each participant was analyzed using the following formula:

$$\mathrm{headmotion}=\frac{\sum_{i=2}^{M}\sqrt{\overset{{\left(x_{i}^{1}-x_{i-1}^{1}\right)}^{2}+{\left(y_{i}^{1}-y_{i-1}^{1}\right)}^{2}+{\left(z_{i}^{1}-z_{i-1}^{1}\right)}^{2}}{+{\left(x_{i}^{2}-x_{i-1}^{2}\right)}^{2}+{\left(y_{i}^{2}-y_{i-1}^{2}\right)}^{2}+{\left(z_{i}^{2}-z_{i-1}^{2}\right)}^{2}}}}{M-1}$$

**TABLE 1:** Demographic characteristics of the two groups.

	**Pilots**		**Controls**		**Significance**	
	**(*N* = 25)**		**(*N* = 24)**			
	***M***	***SD***	***M***	***SD***	***T* value**	***p*-Value**
						**(two-tailed)**
Age (years)	25.92	3.12	29.33	4.02	−3.17	0.003^∗^
Sex (% male)	100%		100%			
Education (years)	16		16			
Handedness (% right)	100		100			
Total flight time (hours)^∗^	430.43	207.90				

*M, mean value; SD, standard deviation. ^∗^The singular values were eliminated.*

*M* is the time point number; *i* represents a certain time point; *x*^1^, *y*^1^, and *z*^1^ are the translations at certain time point; *x*^2^, *y*^2^, and *z*^2^ are the rotations at certain time point.

For the structural scans, the images were segmented into white matter, gray matter, and cerebrospinal fluid. A specific template of this study for normalization was created using the DARTEL toolbox. Subsequently, the functional images were coregistered to the structural images and transformed into standard MNI space (3 mm × 3 mm × 3 mm). Finally, the images were smoothed using 8 mm full-width at half maximum Gaussian kernel.

### Group-ICA

Group ICA is a data-driven method that can identify intercorrelated slow fluctuations in the fMRI signals. GIFT software (version 1.4b), run under MATLAB 2013b, was used to perform spatial ICA (Calhoun et al., 2001). The minimum description length criterion was used to estimate the dimensions of the datasets from the 49 subjects. Thirty-five independent components were ultimately determined. Data from all subjects were concatenated and principal component analysis was used for these datasets to reduce the temporal dimension. The data were decomposed using the Informax algorithm by independent component estimation. This operation was repeated 20 times in ICASSO. The cluster size was set as 16 to get the stable run. Finally, individual subject components were back-reconstructed into single-subject space and the intensity values were scaled to Z scores.

A two-step process was used to select the component that best matched the DMN. First, a frequency filter was applied to exclude any components in which high-frequency signal (>0.1 Hz) constituted ≥50% of the power. Then, the DMN component was selected using an automated template-matching procedure combined by four expert inspection. The components that exhibited close relationship with the DMN template (Luo et al., 2011) and low spatial overlap with known vascular, ventricular, motion and other susceptibility artifacts, were selected.

A two-sample *t*-test was conducted to quantitatively compare the DMN between pilots and controls (GRF correction, *p* < 0.05, with an initial height threshold of *p* < 0.001), controlling for age and head motion effects. This analysis was performed within the mask, which resulted from the one-sample *t*-test of the group (*p* < 0.001).

### Functional Connectivity Analysis

Brain regions that exhibited significant group differences were selected as seeds to further calculate the functional connectivity map. The smoothed images were further regressed using six head motion parameters, global mean signal, white matter signal, cerebrospinal fluid signal, and linear drift signal (Dong et al., 2018). The resulting images were then band-pass filtered (0.01–0.08 Hz) (Fox et al., 2005). Pearson correlation coefficients between time courses of the seeds and of the remainder of the voxels of the entire brain were calculated and Fisher Z transformed. Group differences were obtained using the two-sample *t*-test (GRF corrected, *p* < 0.05, with an initial height threshold of *p* < 0.001) which was performed within the mask obtained from one-sample *t*-test (FDR corrected, *p* < 0.05), controlling for age and head motion effects.

Finally, the relationship between the *Z* value indexes of functional properties and the total flight hours were determined by calculating the Kendall correlation coefficient.

## Results

### Spatial Pattern of Networks

**FIGURE 1:**
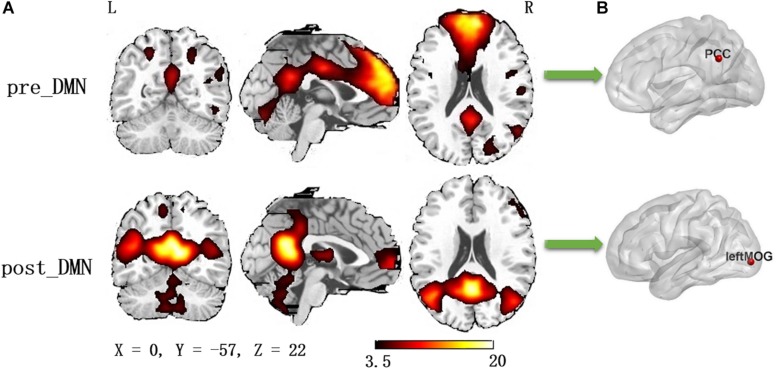
**(A)** The spatial distribution of the default mode network (one-sample *t*-test, *p* < 0.001, uncorrected); **(B)** The pilot group revealed increased functional integration of the precuneus/PCC and left middle occipital gyrus (GRF corrected, *p* < 0.05, the initial height threshold is *p* < 0.001).

According to the automated template-matching procedure and a previous study (Luo et al., 2011), the Z maps of the DMN were identified, including pre_DMN (*r* = 0.172) and post_DMN (*r* = 0.114). The spatial pattern of the components were matched to previous results (Damoiseaux et al., 2006; Mantini et al., 2009). According to the one-sample *t*-test (*p* < 0.001 [uncorrected]), the DMN primarily contained the medial part of superior frontal gyrus, PCC, bilateral angular gyrus, bilateral middle occipital gyrus (Figure 1A and Table 2).

**TABLE 2:** Brain regions with peak value of the DMN.

	**Center**			**Peak**	**Brain regions**
	**(MNI)**			***T* value**	**(AAL)**
	***x***	***y***	***z***		
Pre_DMN	−6	54	21	29.5735	Superior frontal gyrus, medial part
Post_DMN	−6	−57	9	33.0221	Posterior cingulate cortex

Group differences were revealed using a two-sample *t*-test. The pilot group exhibited increased functional integration of the precuneus/PCC and left middle occipital gyrus (left_MOG) (GRF corrected, *p* < 0.05; initial height threshold, *p* < 0.001) (Figure 1B and Table 3).

**TABLE 3:** Brain regions with significant group differences in the DMN (GRF corrected, *p* < 0.05, the initial height threshold is *p* < 0.001).

**Network**	**Center**			**Peak**	**Cluster size**	**Brain regions**
	**(MNI)**			***T* value**	**(voxel number)**	**(AAL)**
	***x***	***y***	***z***			
Pre_DMN	6	−51	33	4.93	20	Right precuneus/Posterior cingulate cortex (PCC)
Post_DMN	−33	−90	−3	5.17	39	Left middle occipital gyrus (left_MOG)

### Functional Connectivity Analysis

**FIGURE 2:**
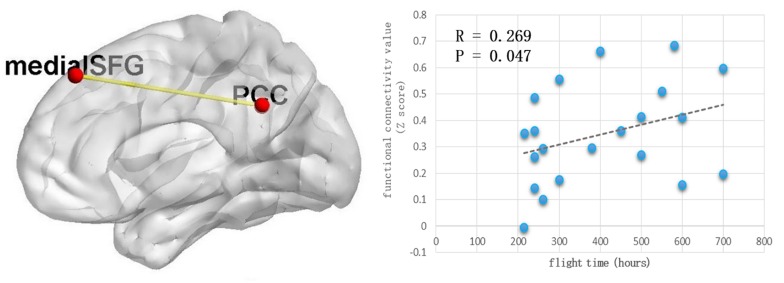
The pilot group exhibited enhanced functional connection between precuneus/PCC and the medial part of superior frontal gyrus (GRF corrected, *p* < 0.05, the initial height threshold is *p* < 0.001). In addition, after eliminating the singular values, this alteration was positively related with the total flight hours.

Because the precuneus/PCC and left_MOG in the DMN exhibited significant group differences, these areas were treated as regions of interest to build the functional connectivity maps for each group. Compared with the control group, the pilot group exhibited enhanced functional connection between the precuneus/PCC and the medial part of superior frontal gyrus (medial_SFG) (GRF corrected, *p* < 0.05; initial height threshold, *p* < 0.001) (Figure 2 and Table 4). The functional connectivity map of left_MOG did not exhibit significant group differences.

**TABLE 4:** Brain regions with significant group differences of functional connection to the seeds (GRF corrected, *p* < 0.05, the initial height threshold is *p* < 0.001).

**Seeds**	**Center**			**Peak**	**Cluster size**	**Brain regions**
	**(MNI)**			***T* value**	**(voxel number)**	**(AAL)**
	***x***	***y***	***z***			
Right precuneus/PCC	6	42	48	3.98	30	Right superior frontal gyrus, medial part (medial_SFG)

After eliminating the singular values, the correlation coefficient was computed between the total flight hours and the *Z* value index of the functional property. Positive correlations were identified (*r* = 0.269, *p* = 0.047) (Figure 2).

## Discussion

The primary aim of the current study was to investigate the functional connectivity pattern of the DMN in pilots. Our results revealed that the pilot group exhibited increased functional integration in the precuneus/PCC and left MOG. Functional connectivity analysis identified enhanced functional connections between the precuneus/PCC and medial SFG. The results suggested a potential relationship between flight experience and functional properties of the DMN, further suggesting that daily flying practice may affect brain function in pilots.

The posterior medial parietal cortex is an associative cortex. The PCC is regarded as the core DMN node, and is involved in maintenance of the conscious state (Leech et al., 2012). In the baseline resting state, the precuneus exhibits the highest metabolic rate among other brain areas in humans (Gusnard and Raichle, 2001). It consumes 35% more glucose than other regions. Meanwhile, it deactivates in many pathophysiological conditions (sleep, the hypnotic state, anesthesia, and vegetative state) and neuropsychiatric disorders, which have been characterized by impaired consciousness (Cavanna, 2007). A widely accepted explanation for this phenomenon is that when an individual is awake and alert, information gathering and representation of the self and external world must be maintained, the precuneus and interconnected PCC are engaged in this continuous process (Gusnard and Raichle, 2001). In addition, the PCC is highly sensitive to the arousal state: when arousal is low, the level of PCC activation is low. PCC is also associated with regulation of focus and the breadth of attention (Leech and Sharp, 2014). During flight, pilots should be alert to the situation, especially take-off and landing because any small mistake or decision-making error may lead to serious consequences. Thus, pilots may exhibit higher levels of arousal at work than other professionals. The increased activity in the PCC of pilots may reflect this tendency. Meanwhile, the MOG is involved in spatial information processing (Renier et al., 2010). It was often detected significant brain activation during active driving (Jeong et al., 2006; Navarro et al., 2018). Increased activation in the MOG of pilots may be due to the large amount of spatial information required for flight.

The functional connections between the precuneus/PCC and medial SFG are increased in pilots. The precuneus and prefrontal cortex are strongly interconnected, and these projections are primarily concentrated at Brodmann areas eight and nine (Cavanna and Trimble, 2006), which are exactly the brain areas we identified. The precuneus/PCC and medial prefrontal cortex are considered to be the core areas of the DMN. This result confirmed that functional connectivity within the DMN is enhanced in pilots and reflected the important role of the DMN in flying aircraft. The DMN is engaged in retrieval and consolidation of episodic memory, conscious representation of information in mental images form (Cavanna and Trimble, 2006). The DMN is also involved in recalling the past, planning the future, and the formation of beliefs (Buckner et al., 2008). Above all, recent studies have identified that the DMN is not simply involved in mind-wandering or recollection, it also plays an important role in externally focused tasks, especially the cognitive transitions (Andrews-Hanna et al., 2010; Sormaz et al., 2018). Andrews-Hanna divided the DMN into three subnetworks, “core,” “medial temporal lobe” and dorsomedial prefrontal area (Andrews-Hanna et al., 2010). During the most between-domain switch trials, the core DMN exhibited increased activity (Crittenden et al., 2015). A correlation between the activation within the DMN and the level of detail in ongoing thought was also identified (Sormaz et al., 2018). It seems like that when there are major cognitive transitions, the DMN activates to encode current, external context by integrating spatial and temporal information to provide related contextual reference (Smith et al., 2018). The DMN encodes internally generated episodes and contexts, it also encodes current external context. Thus, the DMN might play complementary roles in organizing complex, goal-directed behavior (Margulies and Smallwood, 2017). Pilots are always working in complex, dynamic environments. Flying is now not so much a “physical job,” but a high-level cognitive activity. The pilot should be completely aware of all conditions in real time, and be ready to deal with various potential emergencies. These processes include continuous cognitive transitions, which are exactly the function of the DMN. Daily flying practice may activate the pilot’s DMN repeatedly and, ultimately, strengthen its activation level during the resting state.

Flying is analogous to driving. They are all complex activities carried out at high speeds in dynamic environment and require the operators to anticipate environment changes (Navarro et al., 2018). The drivers’ tasks were often categorized in the three levels of skills: strategical, tactical and operational levels; in addition, meta-analysis of neuroscience studies identified that the prefrontal cortex, the occipital cortex and cerebellum were engaged (Navarro et al., 2018). However, flying is also a complex activity, but there is no universal pilot model. In the current study, we identified the potential importance of DMN in flying. The function of DMN may help us to learn more about the flying mechanism. In addition, the increased functional connection within DMN might be a candidate of pilot selection and a biomarker of psychological training level. Meanwhile, more ergonomics and neuroscience studies should be conducted to confirm our findings.

The current study had several limitations. First, the age difference between the two groups may have been a confounding factor. In addition, this study lacked relevant neuropsychological tests to assess cognitive functions of the pilots. As such, explanation of the results could only rely on speculation; therefore, additional assessments should be included in subsequent research. Finally, due to the relatively small sample size, the findings should be confirmed in a larger sample.

## Conclusion

In conclusion, to our knowledge, the current study was the first to consider functional properties of the DMN in pilots. The pilot group exhibited increased functional connectivity within the DMN, especially enhanced functional integration between the anterior (medial_SFG) and posterior (PCC) DMN. These findings highlight the importance of the DMN in the neurophysiological mechanism(s) of flying.

## Data Availability Statement

The raw data supporting the conclusions of this article will be made available by the authors, without undue reservation, to any qualified researcher.

## Ethics Statement

The studies involving human participants were reviewed and approved by the Ethics Committee of University of Electronic Science and Technology of China. The patients/participants provided their written informed consent to participate in this study.

## Author Contributions

XC, KX, YY, and QW designed the experiment, wrote the manuscript, and gave final approval of the version to be published. HJ, XG, and XPC collected and analyzed the data. JY and CL made a substantial contribution to the analysis, interpretation of the data, and critically revised the manuscript.

## Conflict of Interest

The authors declare that the research was conducted in the absence of any commercial or financial relationships that could be construed as a potential conflict of interest.

## Footnotes

1 http://www.fil.ion.ucl.ac.uk/spm/

## References

[B1] Adamson M. M. Taylor J. L. Heraldez D. Khorasani A. Noda A. Hernandez B. (2014). Higher landing accuracy in expert pilots is associated with lower activity in the caudate nucleus. *PLoS One* 9:e112607. 10.1371/journal.pone.0112607 25426935 PMC4245093

[B2] Andrews-Hanna J. R. Reidler J. S. Sepulcre J. Poulin R. Buckner R. L. (2010). Functional-anatomic fractionation of the brain’s default network. *Neuron* 65 550–562. 10.1016/j.neuron.2010.02.005 20188659 PMC2848443

[B3] Buckner R. L. Andrews-Hanna J. R. Schacter D. L. (2008). The brain’s default network: anatomy, function, and relevance to disease. *Ann. N. Y. Acad. Sci.* 1124 1–38. 10.1196/annals.1440.011 18400922

[B4] Calhoun V. D. Adali T. Pearlson G. D. Pekar J. J. (2001). A method for making group inferences from functional MRI data using independent component analysis. *Hum. Brain Mapp.* 14 140–151. 10.1002/hbm.1048 11559959 PMC6871952

[B5] Callan D. E. Terzibas C. Cassel D. B. Callan A. Kawato M. Sato M. A. (2013). Differential activation of brain regions involved with error-feedback and imitation based motor simulation when observing self and an expert’s actions in pilots and non-pilots on a complex glider landing task. *Neuroimage* 72 55–68. 10.1016/j.neuroimage.2013.01.028 23357079

[B6] Causse M. Baracat B. Pastor J. Dehais F. (2011). Reward and uncertainty favor risky decision-making in pilots: evidence from cardiovascular and oculometric measurements. *Appl. Psychophys. Biofeedb.* 36 231–242. 10.1007/s10484-011-9163-0 21739293

[B7] Causse M. Peran P. Dehais F. Caravasso C. F. Zeffiro T. Sabatini U. (2013). Affective decision making under uncertainty during a plausible aviation task: an fMRI study. *Neuroimage* 71 19–29. 10.1016/j.neuroimage.2012.12.060 23313780

[B8] Cavanna A. E. (2007). The precuneus and consciousness. *CNS Spectr.* 12 545–552. 10.1017/s1092852900021295 17603406

[B9] Cavanna A. E. Trimble M. R. (2006). The precuneus: a review of its functional anatomy and behavioural correlates. *Brain* 129(Pt 3), 564–583. 10.1093/brain/awl004 16399806

[B10] Crittenden B. M. Mitchell D. J. Duncan J. (2015). Recruitment of the default mode network during a demanding act of executive control. *eLife* 4:e06481. 10.7554/eLife.06481 25866927 PMC4427863

[B11] Damoiseaux J. S. Rombouts S. A. Barkhof F. Scheltens P. Stam C. J. Smith S. M. (2006). Consistent resting-state networks across healthy subjects. *Proc. Natl. Acad. Sci. U.S.A.* 103 13848–13853. 10.1073/pnas.0601417103 16945915 PMC1564249

[B12] Dong L. Luo C. Liu X. Jiang S. Li F. Feng H. (2018). Neuroscience information toolbox: an open source toolbox for EEG-fMRI multimodal fusion analysis. *Front. Neuroinf.* 12:56. 10.3389/fninf.2018.00056 30197593 PMC6117508

[B13] Dong L. Wang P. Peng R. Jiang S. Klugah-Brown B. Luo C. (2016). Altered basal ganglia-cortical functional connections in frontal lobe epilepsy: a resting-state fMRI study. *Epilepsy Res.* 128 12–20. 10.1016/j.eplepsyres.2016.10.011 27792884

[B14] Durantin G. Dehais F. Gonthier N. Terzibas C. Callan D. E. (2017). Neural signature of inattentional deafness. *Hum. Brain Mapp.* 38 5440–5455. 10.1002/hbm.23735 28744950 PMC6866714

[B15] Fox M. D. Snyder A. Z. Vincent J. L. Corbetta M. Van Essen D. C. Raichle M. E. (2005). The human brain is intrinsically organized into dynamic, anticorrelated functional networks. *Proc. Natl. Acad. Sci. US.A.* 102 9673–9678. 10.1073/pnas.0504136102 15976020 PMC1157105

[B16] Gateau T. Ayaz H. Dehais F. (2018). In silico vs. over the clouds: on-the-fly mental state estimation of aircraft pilots, using a functional near infrared spectroscopy based passive-BCI. *Front. Hum. Neurosci.* 12:187. 10.3389/fnhum.2018.00187 29867411 PMC5966564

[B17] Gong J. Chen G. Jia Y. Zhong S. Zhao L. Luo X. (2019). Disrupted functional connectivity within the default mode network and salience network in unmedicated bipolar II disorder. *Prog. Neuropsychopharmacol. Biol. Psychiatry* 88 11–18. 10.1016/j.pnpbp.2018.06.012 29958116

[B18] Gusnard D. A. Raichle M. E. (2001). Searching for a baseline: functional imaging and the resting human brain. *Nat. Rev. Neurosci.* 2 685–694. 10.1038/35094500 11584306

[B19] Jeong M. Tashiro M. Singh L. N. Yamaguchi K. Horikawa E. Miyake M. (2006). Functional brain mapping of actual car-driving using [18F]FDG-PET. *Ann. Nuclear Med.* 20 623–628. 10.1007/bf02984660 17294673

[B20] Jiang Y. Duan M. Chen X. Zhang X. Gong J. Dong D. (2019a). Aberrant prefrontal-thalamic-cerebellar circuit in schizophrenia and depression: evidence from a possible causal connectivity. *Int. J. Neural. Syst.* 29:1850032. 10.1142/S0129065718500326 30149746

[B21] Jiang Y. Luo C. Li X. Li Y. Yang H. Li J. (2019b). White-matter functional networks changes in patients with schizophrenia. *Neuroimage* 190 172–181. 10.1016/j.neuroimage.2018.04.018 29660513

[B22] Leech R. Braga R. Sharp D. J. (2012). Echoes of the brain within the posterior cingulate cortex. *J. Neurosci.* 32 215–222. 10.1523/JNEUROSCI.3689-11.2012 22219283 PMC6621313

[B23] Leech R. Sharp D. J. (2014). The role of the posterior cingulate cortex in cognition and disease. *Brain* 137(Pt 1), 12–32. 10.1093/brain/awt162 23869106 PMC3891440

[B24] Luo C. Li Q. Lai Y. Xia Y. Qin Y. Liao W. (2011). Altered functional connectivity in default mode network in absence epilepsy: a resting-state fMRI study. *Hum. Brain Mapp.* 32 438–449. 10.1002/hbm.21034 21319269 PMC6870112

[B25] Mantini D. Corbetta M. Perrucci M. G. Romani G. L. Del Gratta C. (2009). Large-scale brain networks account for sustained and transient activity during target detection. *Neuroimage* 44 265–274. 10.1016/j.neuroimage.2008.08.019 18793734 PMC5745806

[B26] Margulies D. S. Smallwood J. (2017). Converging evidence for the role of transmodal cortex in cognition. *Proc. Natl. Acad. Sci. U.S.A.* 114 12641–12643. 10.1073/pnas.1717374114 29142008 PMC5715795

[B27] Musson D. M. Sandal G. M. Helmreich R. L. (2004). Personality characteristics and trait clusters in final stage astronaut selection. *Aviat Space Environ. Med.* 75 342–349. 15086124

[B28] Navarro J. Reynaud E. Osiurak F. (2018). Neuroergonomics of car driving: a critical meta-analysis of neuroimaging data on the human brain behind the wheel. *Neurosci. Biobehav. Rev.* 95 464–479. 10.1016/j.neubiorev.2018.10.016 30442593

[B29] Pauley K. O’Hare D. Wiggins M. (2008). Risk tolerance and pilot involvement in hazardous events and flight into adverse weather. *J. Safety Res.* 39 403–411. 10.1016/j.jsr.2008.05.009 18786427

[B30] Peres M. Van De Moortele P. F. Pierard C. Lehericy S. Satabin P. Le Bihan D. (2000). Functional magnetic resonance imaging of mental strategy in a simulated aviation performance task. *Aviat Space Environ. Med.* 71 1218–1231. 11439722

[B31] Piccardi L. Verde P. Bianchini F. Morgagni F. Guariglia C. Strollo F. (2013). Mental rotation task in a pilot during and after pregnancy. *Aviat Space Environ. Med.* 84 1092–1094. 10.3357/asem.3629.2013 24261064

[B32] Poropat A. E. (2009). A meta-analysis of the five-factor model of personality and academic performance. *Psychol. Bull.* 135 322–338. 10.1037/a0014996 19254083

[B33] Raichle M. E. Snyder A. Z. (2007). A default mode of brain function: a brief history of an evolving idea. *Neuroimage* 37 1083–1090. 10.1016/j.neuroimage.2007.02.041 17719799

[B34] Rangel A. Camerer C. Montague P. R. (2008). A framework for studying the neurobiology of value-based decision making. *Nat. Rev. Neurosci.* 9 545–556. 10.1038/nrn2357 18545266 PMC4332708

[B35] Renier L. A. Anurova I. De Volder A. G. Carlson S. VanMeter J. Rauschecker J. P. (2010). Preserved functional specialization for spatial processing in the middle occipital gyrus of the early blind. *Neuron* 68 138–148. 10.1016/j.neuron.2010.09.021 20920797 PMC2951740

[B36] Sestieri C. Corbetta M. Romani G. L. Shulman G. L. (2011). Episodic memory retrieval, parietal cortex, and the default mode network: functional and topographic analyses. *J. Neurosci.* 31 4407–4420. 10.1523/JNEUROSCI.3335-10.2011 21430142 PMC3098040

[B37] Smith V. Mitchell D. J. Duncan J. (2018). Role of the default mode network in cognitive transitions. *Cereb. Cortex* 28 3685–3696. 10.1093/cercor/bhy167 30060098 PMC6132281

[B38] Sormaz M. Murphy C. Wang H. T. Hymers M. Karapanagiotidis T. Poerio G. (2018). Default mode network can support the level of detail in experience during active task states. *Proc. Natl. Acad. Sci. U.S.A.* 115 9318–9323. 10.1073/pnas.1721259115 30150393 PMC6140531

[B39] Toppi J. Borghini G. Petti M. He E. J. De Giusti V. He B. (2016). Investigating cooperative behavior in ecological settings: an EEG hyperscanning study. *PLoS One* 11:e0154236. 10.1371/journal.pone.0154236 27124558 PMC4849782

[B40] Uddin L. Q. Kelly A. M. Biswal B. B. Castellanos F. X. Milham M. P. (2009). Functional connectivity of default mode network components: correlation, anticorrelation, and causality. *Hum. Brain Mapp.* 30 625–637. 10.1002/hbm.20531 18219617 PMC3654104

[B41] Vannini P. O’Brien J. O’Keefe K. Pihlajamaki M. Laviolette P. Sperling R. A. (2011). What goes down must come up: role of the posteromedial cortices in encoding and retrieval. *Cereb. Cortex* 21 22–34. 10.1093/cercor/bhq051 20363808 PMC3000562

[B42] You X. Ji M. Han H. (2013). The effects of risk perception and flight experience on airline pilots’ locus of control with regard to safety operation behaviors. *Accid Anal. Prev.* 57 131–139. 10.1016/j.aap.2013.03.036 23680497

